# Integrating Crime Prevention and Health Promotion Programs for School‐Aged Children to Decrease Anti‐Social Behaviours

**DOI:** 10.1002/hpja.70085

**Published:** 2025-08-22

**Authors:** Alan Silburn, Kayla Ibrahim

**Affiliations:** ^1^ Western Sydney University Campbelltown Australia

**Keywords:** anti‐social behaviours, crime prevention, health promotion, intervention programs, school‐aged children, socioeconomic factors

## Abstract

The interaction between crime prevention and health promotion programs and their impact on crime rates and antisocial behaviours in school‐aged children is a complex and multifaceted subject. This literature review examines 10 research articles to discern recurring themes, challenges, and potential synergies in addressing these intricate dynamics. It emphasises the crucial role of socioeconomic factors, mental health, and community environments in shaping the effectiveness of interventions. The intersection of crime prevention and health promotion emerges as a promising avenue for comprehensive strategies. However, challenges such as attendance rates and demographic variations underline the need for tailored approaches. The implications for policy, as well as potential directions for future research, are thoroughly discussed.

## 1 Introduction

The journey from childhood to adolescence is marked by challenges, none more significant than the emergence of antisocial behaviours among school‐aged children [1]. While early childhood experiences, including exposure to stress and adversity, play a critical role in shaping the development of antisocial behaviours [1], this review focuses specifically on school‐aged children, an important stage where behavioural patterns consolidate and targeted interventions can effectively alter developmental trajectories. Understanding the impact of crime rates and antisocial behaviours during these formative years is crucial [1]. Such behaviours not only affect individuals but also influence wider community dynamics, necessitating effective interventions. To create lasting positive change, it is essential to comprehend the factors leading to such behaviours, considering socio‐economic, environmental, and mental health aspects [1].

Antisocial behaviours rarely have a single cause. They often result from a complex mix of socio‐economic status, school environment, mental health, and broader community context. While crime prevention and health promotion are usually treated separately, recent research suggests they are interconnected [2]. Approaches that simultaneously address both thus may have more favourable results [2]. This review explores existing research, uncovering how crime prevention and health promotion can work together for more effective outcomes.

Drawing insights from 10 research articles, this review aims to unravel common themes, challenges, and potential synergies in crime prevention and health promotion programmes for school‐aged children. By examining existing literature, the review seeks to inform policies and practices, offering a solid foundation for understanding the dynamics involved. The subsequent sections break down factors influencing antisocial behaviours, the effectiveness of intervention programmes, challenges faced, and the impact of demographic variations. In doing so, it provides a comprehensive overview for shaping evidence‐based interventions suited to the diverse needs of school‐aged children.

Upper primary and middle school‐aged children (approximately 8–12 years old) were chosen as the focus of this review due to their critical developmental stage, where behavioural patterns begin to solidify, peer influences intensify, and autonomy increases. Although early intervention is often defined in the literature as targeting early childhood, this review adopts a definition aligned with the school‐aged years, an age range widely recognised as part of middle childhood in both educational and developmental contexts. Research indicates that interventions during this stage are particularly effective in preventing the escalation of antisocial behaviours into adolescence and adulthood [1]. This age group remains highly responsive to intervention and presents an opportune window for reinforcing positive social behaviours and resilience. Furthermore, the reviewed studies most frequently represented this demographic, reinforcing the relevance of this focus for identifying interventions with lasting individual and community impact.

## 2 Research Question

Crime rates, including theft, violence, delinquency, and drug and alcohol use, are a major concern for the general public. Among the various approaches to managing crime rates, afterschool programs that incorporate crime prevention or health promotion are notably less documented. Understanding the impact of these programs requires examining multiple factors, including those that influence antisocial behaviours, participant engagement, and overall program effectiveness. Similarly, the factors opposing program implementation and utilisation need addressing. Given these considerations, this review seeks to answer the research question:

PICO Question: *In school‐aged children, what is the effect of crime prevention and health promotion programmes on crime rates and antisocial behaviours?*

The term ‘crime rates’ was included in the research question to reflect the broader societal and policy interest in reducing crime; however, the reviewed literature primarily focuses on individual and group‐level changes in antisocial behaviours within various intervention settings.

Although the research question refers to effects on crime rates, it is important to clarify that the evidence reviewed primarily addresses changes in antisocial and related behaviours among participants in various intervention settings, including but not limited to after‐school programmes. The interventions examined span school‐ and community‐based contexts. While these programmes demonstrate promising behavioural outcomes at the individual and group levels, the available literature does not provide direct evidence linking these changes to reductions in crime rates at the population level. Therefore, any broader impact on community crime remains hypothetical and warrants further investigation.

## 3 Search Strategy

For this review, the Ovid Medline Complete and Embase databases were utilised, as both have proven worth producing credible literature covering a wide range of scientific, medical, and healthcare disciplines [3], thus adequate for the proposed research question. Although the review explores both health promotion and crime prevention, the search was conducted primarily through a public health perspective. As such, criminology‐specific databases were not included, which may have limited the capture of some relevant criminal justice literature. Future reviews could benefit from a more interdisciplinary approach that includes both health and criminological sources.

To generate a valid script, the terms need to be contemplated to ensure the results are relevant whilst not being over‐limited. To achieve this, a PICOT framework [4] was used to refine the key components of the study. The search terms used for selecting relevant publications are outlined in Table 1.

**TABLE 1:** PICOT and alternative terms.

Keywords/search terms/phrases		Alternative words/terms considered
P	Children, aged 8–12	Child/aged 8–12/preteen/middle childhood/primary school
**I**	Crime prevention programs	Crime prevention/health promotion/afterschool
**C**	No programs	Nil
**O**	Crime rates	Crimes rates/antisocial behaviour/truancy/school attendance rates/thief/violence/drug use/alcohol use/cybercrime
**T**	Between 2004–2024	20 040 201–20 240 201; since 2004

Once selected, search terms were either combined with truncation methods and Boolean operators or controlled with Medical Subject Headings in a strategic means to produce relevant results for possible inclusion. This resulted in the following search action:

((child* OR ‘primary school student*’ OR preteen*) AND (‘*Crime prevention*’ *OR* ‘*health promotion*’ *OR* ‘*after school*’*) AND program AND (Crime OR truancy OR* ‘*school attendance*’ *OR theft OR violence OR* ‘*drug use*’ *OR* ‘*alcohol use*’*)).af*.

## 4 Inclusion and Exclusion Criteria

This study aims to determine the effect of crime prevention and health promotion programmes on crime rates and antisocial behaviours. Articles were considered for inclusion if they met the criteria outlined in the PICOT framework, remained after the screening process, and did not meet any exclusion criteria.

**FIGURE 1:**
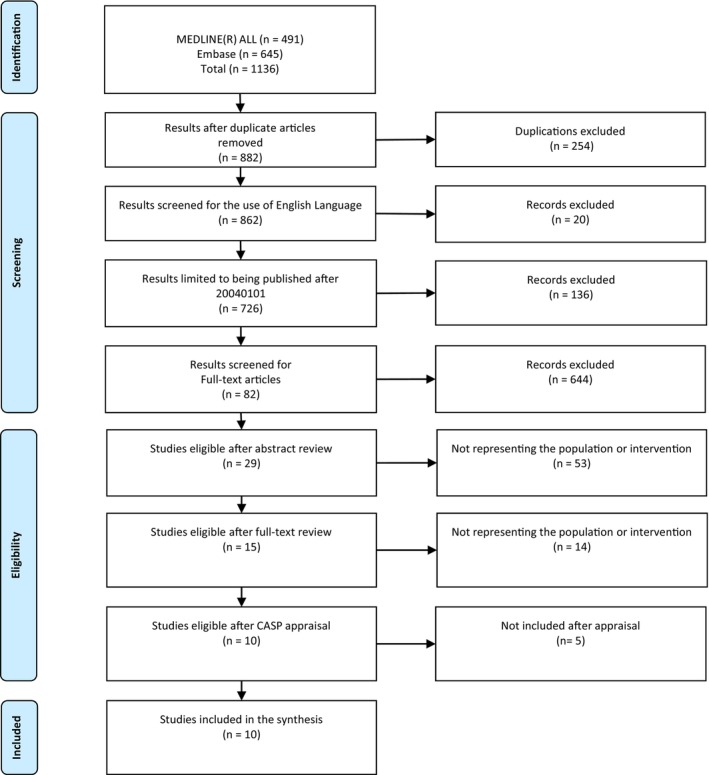
PRISMA diagram.

After the preliminary search, article duplicates were identified and removed. The screening was possible by using the search engine limiters for literature published in the English language and published since 1 January 2004 to ensure currency. Although 10 years is preferred regarding timeframe, this broader timeframe accounts for the longitudinal nature of many studies, which assess behavioural outcomes over extended periods from childhood through adolescence. Additional screening was conducted limiting to full‐text academic journals to ensure quality. The first‐pass exclusion was conducted by assessing the article's abstract for comparability, eliminating any non‐comparable intervention or population characteristic. Second‐pass exclusion occurred by assessing the full article, ensuring that crime or antisocial behaviours were identifiable. Of the articles remaining, all were assessed for quality and potential bias using the Critical Appraisal Skills Programme [5] appraisal methodology. This process is further displayed in Figure 1 PRISMA diagram [6].

## 5 Results

The articles included display similar attributes worthy of inclusion, whilst also being different in research design, population demographics, and intervention modality. Table 2 further displays the characteristics of the literature.

**TABLE 2:** Characteristics of the literature.

Title	Year, country	Authors	Target population and behaviour	Focus and intervention type	Delivery setting	Study design	Outcomes and relevance to research question
Comparative effectiveness of an economic empowerment program on adolescent economic assets, education and health in a humanitarian setting	2020, Democratic Republic of the Congo	Glass et al.	Adolescents in humanitarian settings; economic independence	*Health focused* Economic empowerment	Humanitarian setting	Mixed methods approach and qualitative interviews with a sub‐sample of young adolescents participating in RFR	Increased financial literacy, school retention, and health outcomes—potential indirect reduction in crime/antisocial behaviour.
Effect of childhood nutrition counselling on intelligence in adolescence	2017, Brazil	Munhoz et al.	School‐aged children; cognitive function and behaviour	*Health focused* Health promotion	School‐based	15‐year longitudinal study	Improved cognitive development—potential protective effect against antisocial behaviour but no direct crime‐related outcomes.
Efficacy evaluation of the school program Unplugged for drug use prevention among Brazilian adolescents	2016, Brazil	Sanchez et al.	Adolescents; drug use prevention	*Crime focused* School‐based prevention	Schools	RCT	Reduced substance use—a known risk factor for crime—suggests potential crime prevention benefits.
Free breakfasts in schools: Cluster RCT of the Primary School Free Breakfast Initiative in Wales	2007, United Kingdom (Wales)	Moore et al.	Primary school children; nutrition and behaviour	*Health focused* School‐based nutrition program	Schools	Cluster RCT and embedded qualitative aspect to the evaluation which incorporated questionnaires, semi‐structured interviews and case studies.	Improved student engagement and behaviour in school—potential reduction in antisocial behaviour.
Helping boys at‐risk of criminal activity: Qualitative results of a multi‐component intervention	2011, Canada	Lipman et al.	At‐risk boys; preventing criminal activity	*Crime focused* Behavioural intervention	Community‐based	Qualitative process evaluation of the intervention program using semi‐structured interviews and focus groups.	Improved social skills, emotional regulation—suggests potential for reduced criminal behaviour.
Longitudinal effects of Youth Empowerment Solutions: Preventing youth aggression and increasing prosocial behaviour	2022, United States of America	Thulin et al.	Youth; aggression reduction and prosocial behaviour	*Integrated focused* Youth empowerment	Community‐based	Longitudinal study	Increased prosocial behaviour, reduced aggression—supports crime prevention through positive social engagement.
Mental health promotion and socio‐economic disadvantage: Substance abuse, violence, crime prevention and child health	2007, Australia	Toumbourou et al.	Disadvantaged youth; mental health and violence prevention	*Integrated focused* Mental health and violence prevention	Community and school	Review	Addresses multiple risk factors for crime—supports integrated approaches for prevention.
Reaching the hard to reach: Adolescent attendance at an after‐school sexual and reproductive health programme in South Africa	2015, South Africa	Mathews et al.	Adolescents in South Africa; health education and behaviour	*Health focused* After‐school education	Community‐based	Longitudinal study	Increased school engagement, potential reduction in risky behaviour—limited direct crime‐related outcomes.
Sport involvement, sport violence and health behaviours of Greek adolescents	2004, Greece	Papaioannou	Greek adolescents; sports and aggression	*Crime focused* Sports‐based intervention	Schools and community sports programs	Cross‐sectional study	Mixed results—some sports reduced antisocial behaviour, others increased aggression.
The Influence of Neighbourhood Crime on Increases in Physical Activity during a Pilot Physical Activity Intervention in Children	2016, United States of America	Broyles et al.	Children in high‐crime neighbourhoods; physical activity	*Health focused* Physical activity promotion	Community‐based	Pilot intervention study	Increased activity but exposure to crime risks—highlights the need for safer environments.

## 6 Discussion

### 6.1 Factors Influencing Antisocial Behaviours

An overarching theme in the literature is the acknowledgment of diverse factors influencing antisocial behaviours in school‐aged children. These factors diffuse across the familial, peer, community, and environmental domains. Understanding these factors is fundamental for designing interventions that address the root causes of antisocial behaviours.

The research defines early intervention as occurring during the early childhood to pre‐adolescent stages, commonly referred to as the ‘school‐aged' years, when children are in a highly influential phase of development. Studies have reported on the timely correlation between antisocial behaviour exposure and increases in challenging behaviours displayed by the child [7, 8]. Children who experience or are exposed to familial domain factors, such as domestic violence, parental incarceration, and familial drug and alcohol use, or peer domain factors, such as peer rejection, bullying, and associations with antisocial peers, are at increased likelihood of displaying negative reactivity traits in childhood [7, 8]. Negative reactivity refers to the inclination of children to respond to stressors with negative emotions, such as anger, fear, difficulty, and irritability [9]. Similarly, perceived role models, such as parents, teachers, community leaders, or peers, can further reinforce antisocial behaviour development by condoning acts, through poor supervision, or inconsistent discipline. As such, the timing of intervention implementation holds paramount importance. The body of research suggests that initiating interventions earlier yields greater improvements in a child's behaviour both at home and at school, consequently leading to a higher likelihood of averting future criminal activity [7, 8, 9].

Regarding the community and environmental realms, lower socioeconomic status (SES) consistently emerges as a pivotal determinant of shaping behaviour, with numerous studies indicating a correlation between low SES communities, quality of school environments, and an increased vulnerability to engaging in antisocial activities [8, 10, 11, 12, 13]. Interventions targeting the root causes of low socioeconomic status demonstrate notable effectiveness in reducing adolescent crime and violence, as well as improving child health outcomes. For instance, Mathews et al. evaluated an after‐school sexual and reproductive health programme in the Western Cape, South Africa, aimed at reaching underserved adolescents through community‐based facilitators and structured group sessions [14]. Broyles et al. piloted a neighbourhood‐based physical activity intervention for children, exploring how local crime rates impacted participation, thus highlighting the intersection of environmental safety and intervention uptake in low SES areas [13]. The program described by Lipman et al. combined mentorship, academic support, and family engagement for boys at risk of criminal involvement in Canada, with qualitative results suggesting improved self‐regulation and reduced behavioural issues [8, 11]. Thulin et al. assessed a program in the US, which used community projects and leadership training to foster prosocial behaviour and reduce aggression among socioeconomically disadvantaged youth [11, 12]. Toumbourou et al. discussed a multi‐pronged health promotion approach in Australia that integrated substance abuse prevention, violence reduction, and child health initiatives, with an emphasis on tailoring strategies to the needs of socioeconomically disadvantaged populations [12].

### 6.2 Intersection of Crime Prevention and Health Promotion

This paragraph outlines the primary methods and reported outcomes of crime prevention programmes aimed at reducing antisocial behaviour in school‐aged children. Aggression, theft, delinquency, and other antisocial behaviours have been identified as precursors to criminal offences [8]. Fortunately, studies have underscored that a reduction in criminal offending among school‐aged children is inducible following the implementation of programmes that increase social progression and competence [7, 8, 10, 11, 12, 15]. Intervention approaches primarily focus on programmes that target family dynamics, peer relationships, and community engagement to reduce antisocial behaviour. These programmes often target specific risk factors such as family instability or high levels of neighbourhood crime and include broader concepts of improvement in family dynamics, relationship building, positive mentorship, and to a lesser extent independence. These types of interventions are effective as they provide both immediate behavioural support and long‐term family‐oriented strategies that reduce the likelihood of criminal behaviours emerging [8, 10, 15]. Additionally, mentoring programmes that focus on positive role models and relationship building also showed significant reductions in delinquent behaviours [8, 11]. One programme reported a highly predictive model post a 12‐month intervention programme encompassing psychological empowerment and a dramatic reduction in aggression episodes and improved prosocial behaviours [11]. Likewise, a study noted a reduction in youth episodes of alcohol intoxication by 20%, the chances of frequent alcohol intoxication by 38%, and the chances of recent cannabis use by 26% across the study groups post‐program involvement [15].

In contrast, the following paragraph examines health promotion initiatives that, while not directly designed to prevent crime, contribute to improved behavioural and mental health outcomes that may indirectly reduce antisocial conduct. Moreover, the literature reports positive outcomes regarding the impact of health promotion programs on mental health, overall well‐being, and the improvement of prosocial behaviours. Health promotion initiatives often target areas of health disparity. In the context of school‐aged children, a social gradient is reportable as children of lower SES families had higher incidences of disclosing skipped meals than their wealthier counterparts. Not eating breakfast has been associated with a wealth of detrimental health outcomes; notably, the effects on memory and concentration [10]. Concerningly, it was reported in a United Kingdom population that 5% of children reported having not had anything for breakfast that day, 3% had only consumed a drink, and approximately a further 10% reported eating crisps or chocolate for breakfast [16]. Thus, a breakfast programme was developed to provide healthy breakfast options to school students in a before‐school program to increase academic performance and improve mental health outlooks. Though not directly designed to reduce crime, the positive outcomes from the breakfast program, including improvements in concentration, mental health, and greater social engagement, likely contributed to a decrease in antisocial behaviour.

Similar to nutrition, sexual health also displays a social gradient. The onset of sexual intercourse at younger ages remains a concern in public health. Associated with problems in health and social development, such as increased likelihood of sexually transmitted infections, substance use, and unintended pregnancies [17], after‐school programmes can educate and improve the health of school‐aged children. The literature suggests that after‐school programmes have had positive effects on academic, social and emotional outcomes with increases in the mean age when intercourse is initiated [14].

Programs delivered through schools and community organisations tend to have a wider reach and benefit from a more integrated approach, while after‐school programs reinforce learned behaviours and offer additional mentorship [12, 14]. Whilst the delivery setting and content are variable, the integration of these intervention methods has been shown to have a positive influence on student outcomes, emphasising the interconnected nature of physical and mental health with behavioural outcomes [11, 12, 16]. These findings suggest that holistic approaches that address both physical and mental well‐being are essential for promoting positive behaviours [11, 12, 15, 18]. Unfortunately, at present, there is no discernible trend in the literature regarding the observation of the intersection between crime prevention and health promotion programs. Despite a clear trend, there is a suggestion in the literature that interventions tackling both crime prevention and health promotion simultaneously may offer more favourable and lasting outcomes, acknowledging the holistic needs of individuals and communities [11, 12].

Taken together, the crime prevention and health promotion programmes reviewed share a core conceptual overlap: both aim to foster the development of personal capabilities that support prosocial, adaptive behaviours. While crime prevention programmes may appear behaviourally corrective, they primarily function by cultivating positive traits, such as emotional regulation, social competence, and relationship‐building, that reduce the space for antisocial behaviours to emerge. Similarly, health promotion initiatives, though often designed to address physical or mental health disparities, promote the same foundational skills and resources necessary for behavioural self‐regulation and positive social engagement. In both domains, the focus shifts from the suppression of ‘bad’ behaviours to the active development of protective factors and supportive environments. This shared emphasis, sometimes extending to families and peer networks, underlines the value of integrating crime prevention and health promotion strategies under a broader agenda of capacity building for youth well‐being.

### 6.3 Challenges and Limitations

While the literature reports positive outcomes, it also candidly discusses challenges and limitations. Low attendance rates, particularly among vulnerable populations, pose a significant hurdle to program success [7, 8, 15, 17]. High attendance and active participation are essential for program success. Strategies to enhance engagement, particularly among vulnerable populations, need to be a focal point in program design [19]. Although universally applicable, the literature emphasises the importance of tailored approaches that consider the unique challenges presented by different communities and social networks. Addressing barriers such as transportation, scheduling conflicts, and community‐specific challenges is paramount. The literature suggests that interventions need to incorporate creative and flexible approaches to overcome attendance challenges, ensuring that the intended beneficiaries, in this instance school children, actively participate in and benefit from the programs [19].

Also, demographic variations and disparities in program effectiveness underscore the need for tailored strategies to maximise impact. It is noted that program assimilation efficacy is often not duplicable across all communities or child groups; hence, demographic adaptation is pertinent to overcome these challenges. Understanding these challenges is crucial for refining program design and implementation to ensure inclusivity and equitable outcomes [18]. Considerations for addressing these challenges should be surmised and actionable following stakeholder engagement and needs analysis [20].

### 6.4 Impact of Demographic Variations

The impact of interventions appears to be influenced by age and gender patterns. Tailoring programmes to specific demographic groups, considering developmental stages, and addressing gender‐specific needs are crucial for engagement and optimal outcomes. The literature suggests that a one‐size‐fits‐all approach may not be effective; necessitating a more targeted and personalised strategy to accommodate the diverse needs of different age groups and genders to better reduce problematic behaviours [19].

The broader community and environmental context significantly influence the success of interventions. Understanding the unique challenges posed by different environments is vital for program design and implementation. Programs need to be sensitive to the socio‐cultural dynamics of the communities they serve [20]. The literature highlights the importance of community engagement, considering local factors, and fostering collaboration between stakeholders to create environments conducive to positive behavioural outcomes [7, 14, 15, 16].

## 7 Conclusions

In summary, the literature on factors influencing antisocial behaviours among school‐aged children highlights the multifaceted nature of these behaviours, encompassing familial, peer, community, and environmental domains. Children exposed to factors such as domestic violence, peer rejection, or low socioeconomic status are more likely to exhibit negative reactivity traits, emphasising the importance of early intervention. Interventions targeting root causes, including family dynamics and neighbourhood crime, show promise in reducing antisocial activities and improving mental health outcomes.

The intersection of crime prevention and health promotion initiatives underscores the potential for holistic approaches to yield positive results, with programs focusing on social progression, family dynamics, and mentorship demonstrating effectiveness. Health promotion efforts, such as breakfast programmes and after‐school initiatives, also contribute to improved academic, social, and emotional outcomes. However, challenges such as low attendance rates and demographic variations in programme effectiveness necessitate tailored strategies and community engagement for inclusivity and equitable outcomes.

Demographic factors, including age and gender, influence program impact, highlighting the need for personalised approaches. Understanding the socio‐cultural dynamics of communities and fostering collaboration among stakeholders is essential for creating supportive environments conducive to positive behavioural outcomes.

While targeted interventions address immediate risk factors like family instability and neighbourhood crime, these often stem from deeper structural inequalities. Socioeconomic disadvantage is a root cause in itself, shaped by broader social and political forces. Sustainable change requires not only localised programmes but also systemic action to reduce inequality through reforms in housing, education, income support, and access to services.

## 8 Implications for Policy and Practice

Policy recommendations include integrating crime prevention and health promotion into standard school curricula, emphasising early intervention, and addressing socio‐economic disparities. Tailored strategies for diverse demographics, continuous programme evaluation, and adaptive implementation are crucial for successful policy implementation. Policymakers need to consider the multifaceted nature of antisocial behaviours and create policies that address both the root causes and the symptoms to foster positive, long‐term change.

## 9 Areas for Future Research

Future research directions should prioritise longitudinal studies, diverse intervention strategies, and the sustained impact of programs over time. A contextualised understanding of the relationships between health and crime program components and outcomes will contribute to the refinement of interventions for optimal effectiveness. Exploring the potential for community‐based interventions and assessing the long‐term societal impact of interventions are areas that warrant further investigation. Further inquiry into school‐aged children is clear; however, considerations may include extending towards high school ages. The literature review provides a foundation for future research, offering insights into potential gaps and avenues for expanding our understanding of effective interventions for school‐aged children.

## Author Contributions

The authors solely contributed to the conception, design, analysis, and drafting of the manuscript.

## Ethics Statement

This study did not require ethical approval as it involved a retrospective analysis of publicly available and anonymized information, with no direct involvement of human subjects.

## Consent

The authors consent to the publication of this article.

## Conflicts of Interest

The authors declare no conflicts of interest.

## Data Availability

Data sharing is not applicable to this article as no new data were created or analyzed in this study.
